# First evaluation of alkylpyrazine application as a novel method to decrease microbial contaminations in processed meat products

**DOI:** 10.1186/s13568-018-0583-6

**Published:** 2018-04-03

**Authors:** Matthias Schöck, Stefan Liebminger, Gabriele Berg, Tomislav Cernava

**Affiliations:** 1 ACIB GmbH, Petersgasse 14, 8010 Graz, Austria; 2 Institute of Environmental Biotechnology, Graz University of Technology, Petersgasse 12, 8010 Graz, Austria; 3 Roombiotic GmbH, Stremayrgasse 16, 8010 Graz, Austria

**Keywords:** Alkylpyrazines, Spoilage bacteria, Antimicrobial volatiles, Volatile organic compounds, Preservation

## Abstract

Every year about 20% of the globally produced meat gets lost due to microbial spoilage. Nevertheless, the demand for processed meat is constantly rising and producers are searching for novel strategies to reduce microbial contaminations in their products. In the present study, we evaluated the applicability of alkylpyrazines as antimicrobial agents. These fragrant molecules naturally occur in different vegetables, fruits, roasted nut and meat. Several pyrazine derivatives are readily added to processed products for flavoring purposes in the food industry. To evaluate their potential for application, two derivatives were tested for their antimicrobial activity against meat-associated bacterial contaminants and chicken meat as a whole. Isolates assigned to *Carnobacteriaceae*, *Enterobacteriaceae*, *Listeriaceae*, and *Moraxellaceae* were substantially inhibited in the pilot tests. Moreover, treatments of pyrazine-susceptible isolates resulted in 4-log reductions in bacterial cell counts. The effect was more pronounced when the model contaminants were exposed to higher concentrations of 5-isobutyl-2,3-dimethylpyrazine. In a first small-scale application with processed chicken meat, it was demonstrated that the antimicrobial effects of 2-isobutyl-3-methylpyrazine can be improved by additionally lowering the water activity on the meat surface when maltodextrin is used as a carrier substance. At low pyrazine dosages, the number of viable bacteria was decreased up to 95% in comparison to the corresponding controls. A complementary imaging method that was developed to assess the efficacy on the product, reinforced the applicability of this two-component system.

## Introduction

Meat is an important energy, protein, and micronutrient source in the human diet. Due to population growth, but also adaption of developing countries, especially China, to the western civilization, the meat demand is steadily increasing (OECD-FAO 2017). The high amount of meat consumption is an enormous burden for the environment nowadays, mainly because it is fastidious in production. It is well known, that animal products generally have a higher water demand than crop products. Depending on the meat source the amount of required water varies from 4300 to 15,400 m^3^/ton (Mekonnen and Hoekstra 2012). In addition to the enormous demands of natural resources for the production of meat, the preventive use of antibiotics made livestock farms a prevailing source of drug-resistant microorganisms (Heuer et al. 2011). Both, the enormous environmental burden of animal protein and the health implications of intensive farming are alerting. Nevertheless, a considerable proportion of produced meat is wasted nowadays. A recent study of the Food and Agriculture Organization of the United Nations (FAO) showed that almost 20% of the whole meat production is lost annually, this conforms to 263 million tons of meat (FAO 2011). Lipid oxidation, autolytic enzymatic spoilage and microbial spoilage are the main causes for spoilage. They are responsible for the change of color, texture, odor and flavor of meat. Microorganisms are an important cause for such deterioration processes (Zhou et al. 2010). To preserve meat, which provides favorable growth condition for different microorganisms, our ancestors invented different techniques like salting, drying, smoking, fermentation and canning (Dave and Ghaly 2011). All of these traditional techniques extent the shelf life of meat and meat products but underlie the disadvantage that they change composition, appearance, tenderness, flavor, juiciness and nutritive value of the treated meat. Nowadays other techniques like refrigeration, ionizing radiation, chemical preservation, high hydrostatic pressure and packaging are more common (Dave and Ghaly 2011; Zhou et al. 2010). They provide the means for better conservation of the physical and chemical properties of fresh meat. Today’s health awareness of consumers led to new trends in food processing (Bigliardi and Galati 2013). Chemical or artificial substances like nitrites, sulphites or benzoic acid are avoided by many consumers (Dave and Ghaly 2011). Alternative conservation techniques like biopreservation and natural antimicrobials are essential for green label products that attract consumers with their natural image (Zhou et al. 2010). Distinct metabolites from microbial origin, including lactic acid, are known to enhance the shelf life of fresh meat (Stiles 1996). Nevertheless, there is still a great demand for efficient and environmentally friendly strategies.

In order to find suitable alternatives for conventional food preservatives, we focused on microbial volatile organic compounds (mVOCs), which are mediators of various interactions in nature (Effmert et al. 2012; Kanchiswamy et al. 2015). They are produced by the majority of known organisms and can serve as communication molecules between organisms of the same species, but also between organisms across organismal kingdoms (Effmert et al. 2012; Ryu et al. 2003). Recently it was shown that alkyl-substituted pyrazines, distinct mVOCs produced by *Paenibacillus polymyxa*, are mediators of antimicrobial effects against a series of plant and human pathogens (Cernava 2012; Rybakova et al. 2016). Specific pyrazine derivatives were already employed for the decontamination of eggshells and demonstrated a comparable efficiency to conventional treatments (Kusstatscher et al. 2017). Pyrazines belong to the chemical group of diazines, which consist of an aromatic heterocyclic ring with two nitrogen atoms in para-orientation and differ in residues substituted to the carbon atoms of the ring structure. By decision of the Flavor and Extract Manufacturers Association (FEMA), pyrazines are declared to receive GRAS status. They are generally recognized as safe in their application as flavoring substances (Adams et al. 2002). Based on these findings, 5-isobutyl-2,3-dimethylpyrazine (5IB23DMP) and 2-isobutyl-3-methylpyrazine (2IB3MP) were utilized as model pyrazines for laboratory scale treatments of processed chicken meat strips. The first substance, 5IB23DMP was used to identify pyrazine sensitive meat-derived microorganisms due to the similar antimicrobial effect to the pyrazine mixture emitted by the highly antagonistic *P. polymyxa* GnDWu39 (Cernava 2012; Rybakova et al. 2016). The second substance, 2IB3MP was used due to its convenient flavor profile, resembling the smell of bell peppers (Rognon and Chastrette 1994). The overall aim of this study was to determine if microbial contaminations can be sufficiently reduced in meat products by application of antimicrobial, alkyl-substituted pyrazines. Moreover, we wanted to define the required dosages for adequate pyrazine treatments that could later find application in meat processing. The potential preservatives were tested in a hurdle technology combining 2IB3MP as active ingredient with maltodextrin as carrier substance and storage at 4 °C. Treatment efficiencies were assessed with cultivation-dependent methods in combination with a novel textile imprint method coupled with confocal laser scanning microscopy.

## Materials and methods

### Antimicrobial efficiency tests with 5-isobutyl-2,3-dimethylpyrazine

In vitro tests with isolated microorganisms were performed in order to access the antimicrobial activity of alkylpyrazines in combination with different bacterial meat contaminants. For the isolation of representative aerobic bacteria various cooked ham products were obtained from local retailers (Graz, Austria). A total amount of 12–15 g of each product was homogenized for 3 min with 50 ml of 0.85% NaCl in a BagMixer (Interscience, France). Following plating on nutrient agar (NA; Carl Roth, Germany) and purification of the colonies, all morphologically distinct isolates were sent for Sanger sequencing of the 16S rRNA gene fragment (LGC Genomics, Germany). The sequences were assigned with BLASTn to database entries with the highest sequence homology (NCBI; https://blast.ncbi.nlm.nih.gov). All representative isolates were then treated with 5-isobutyl-2,3-dimethylpyrazine (5IB23DMP) to evaluate its antimicrobial efficiency. Aqueous pyrazine solution containing 0.3 and 0.6% 5IB23DMP were inoculated with overnight cultures of the isolates that were diluted to an OD_600_ = 0.07. The suspensions were incubated for 4 h at 30 °C and continuous shaking at 130 rpm. Finally, the suspensions were plated on NA plates and the reduction of viable cells was determined by comparisons with non-treated controls. For taxonomic identifications, 16S rRNA gene fragments were amplified with the 27F/1492R primer pair. All 16S rRNA gene fragment sequences were deposited at Genbank (https://ncbi.nlm.nih.gov/genbank).

### Combined treatments of processed meat with 2-isobutyl-3-methylpyrazine and maltodextrin

The processed chicken meat strips, which were, according to manufacturer’s information, roasted in sunflower oil and salted with table salt, were obtained from a local supermarket (Graz, Austria). All samples utilized for this study were packed in sealed plastic containers. A mixture of maltodextrin (Myprotein, UK) containing 20% 2-isobutyl-3-methylpyrazine (2IB3MP; FCI SAS, France) was utilized as active ingredient for the treatments. The weight of the meat strips was determined in sterile 50 ml plastic tubes and then stored on ice during further processing. Three distinct amounts of active ingredient (0.015 g/g meat, 0.045 g/g meat and 0.075 g/g meat) were added. After addition of the active ingredient the tubes were shaken for 15 min on an automated shaker at 130 rpm to ensure homogeneous distribution on the meat pieces. The tubes were incubated for 48 and 96 h at 4 °C to simulate storage conditions. To verify the efficiency of the treatment, meat strips together with 10 ml of 0.85% NaCl for each g meat were added into flasks and shaken for 15 min at 130 rpm to detach the microorganisms. The resulting suspension was diluted and plated on NA plates. After incubation of the plates at room temperature for 48 h the colony forming units per gram (CFU/g) meat were calculated. In addition, a control without maltodextrin and a second control containing pure maltodextrin (0.036 g/g meat) without 2IB3MP was incubated with the treated samples. All quantifications of viable cells were conducted with five replicates for each treatment and sampling time point.

### Analysis of textile imprints with differential staining

**Fig. 1:**
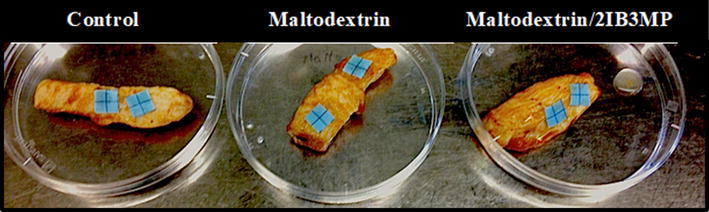
New imprint method for the visualization of microbial contaminations on processed meat strips. The textile pieces were placed onto the meat pieces after they were incubated for 72 h at 4 °C and were then incubated for additional 24 h before differential staining and confocal microscopy

Polyester-based textile fabrics used for the production of clean room garments (Dastex, Muggensturm, Germany) of a defined size (1 cm × 1 cm) were excised. The textile pieces were autoclaved and dried at 65 °C. Chicken meat pieces were treated with maltodextrin (0.036 g/g meat) or maltodextrin/2IB3MP (0.075 g/g meat). Controls were included, these samples were not treated with any of the active ingredients. The meat pieces were stored for 72 h at 4 °C. After storage, two textile pieces were attached to each piece of meat (Fig. 1) and stored at 4 °C for additional 24 h. The textile pieces inoculated on the meat were stained with the LIVE/DEAD^®^ Baclight™ kit (Invitrogen, Karlsruhe, Germany). The dyes were prepared as described in the manufacturer’s protocol. A final volume of 100 μl of each fluorochrome solution was transferred onto the textiles and incubated for 15 min in the dark. The micrographs were recorded with a Leica TCS SPE confocal laser-scanning microscope (Leica Microsystems, Mannheim, Germany). For the SYTO^®^ 9 green-fluorescent dye an extinction/emission wavelength at 485/530 nm was applied and for the red-fluorescent dye propidium iodide an extinction/emission wavelength at 485/630 nm.

### Statistical analysis

For the microbial reduction experiments, the statistical analyses (ANOVA and post hoc analysis with Scheffe’s test) were performed with Data Analysis ToolPak StatPlus (AnalystSoft Inc., Los Angeles, USA).

## Results

### Treatment of isolated meat contaminants with 5-isobutyl-2,3-dimethylpyrazine

The preliminary experiments employed for the antimicrobial efficiency evaluations of 5IB23DMP showed a substantial reduction of livable cells in five out of the eight tested isolates in 0.3% aqueous suspension (Table 1). In 0.6% pyrazine suspensions, a log-4 reduction was observed for one additional isolate that was not affected by the lower concentration. Isolates that were not affected by the employed pyrazine derivative were assigned to the bacterial lineage *Bacillus* at genus level. One of these resilient isolates was assigned to *Bacillus amyloliquefaciens*, while the other was not assignable at species level by analysis of the 16S rRNA gene fragment. The isolate that was able to withstand 0.3% 5IB23DMP treatments was identified as *Carnobacterium maltaromaticum*, which belongs to the Gram-positive *Firmicutes*. Three of the sensitive isolates were assigned to *Gammaproteobacteria*, with two members of the bacterial family *Enterobacteriaceae*. *Brochothrix thermosphacta*, a bacterium associated with meat spoilage, which belongs to the Gram-positive *Listeriaceae* was also substantially reduced at both tested pyrazine concentrations.

**Table 1:** Efficacy of an alkylpyrazine derivative (5-isobutyl-2,3-dimethylpyrazine) against bacteria

Tested isolate	4-log reduction in aqueous pyrazine solution	
	0.3%	0.6%
*Bacillus amyloliquefaciens* HT1C	−	−
*Bacillus* sp. HT1B	−	−
*Brochothrix thermosphacta* HT1G	+	+
*Carnobacterium maltaromaticum* HT1Z	−	+
*Proteus* sp. HT1W	+	+
*Psychrobacter* sp. HT1F	+	+
*Serratia* sp. HT1E	+	+
*Sphingobacterium faecium* HT1X	+	+

Pure cultures of isolates obtained from processed meat products were incubated in aqueous pyrazine solutions. Combinations of isolates and pyrazine concentrations that led to a 4-log reduction are labeled with ‘+’, while such with lower reduction rates are labeled with ‘−’

### Quantification of aerobic mesophilic bacteria on pyrazine-treated meat samples

**Fig. 2:**
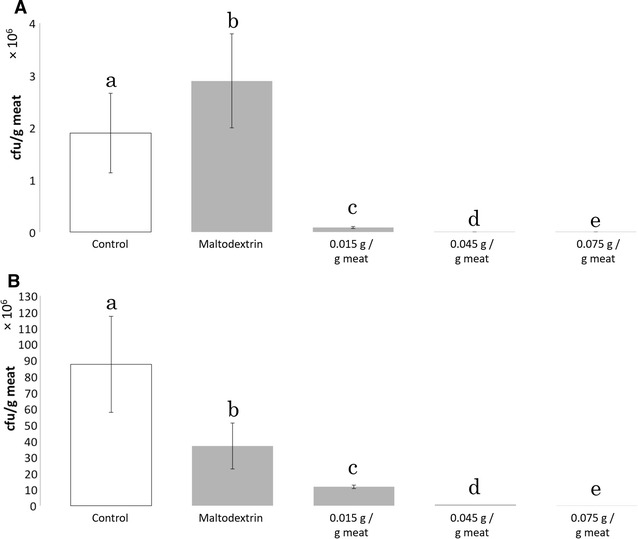
Quantification of livable bacteria following storage at 4 °C. The meat strips were incubated in a refrigerator for 24 h (**A**) and 96 h (**B**). Following homogenization of the sample, dilution and plating, the plates were incubated for 48 h at 21 °C before quantification of CFU/g meat. Different letters denote significant differences among the tested concentrations (P < 0.05)

The processed chicken meat strips were treated with three defined dosages of maltodextrin with 20% 2IB3MP (w/w) and compared to a non-treated control. In addition, processed meat samples were treated with maltodextrin without pyrazine supplementation (0.045 g/g meat). Treatments with pure maltodextrin that was not supplemented with pyrazine, showed a significant reduction in CFU counts after 96 h of storage (Fig. 2). Here, the treated samples had an average of 3.7 × 10^7^ CFU/g, while the control contained 8.75 × 10^7^ CFU/g. Significant reductions in viable bacteria occurred already after 48 h in all pyrazine-containing samples (Fig. 2). Dosage-dependent reductions were observed when different maltodextrin/2IB3MP dosing was compared. Following the storage at 4 °C and 48 h, samples that were treated with 0.015 g of the antimicrobial preparation per Gramm meat, contained 8.65 × 10^4^ CFU/g compared to 5.7 × 10^2^ CFU/g for the highest dosage (0.075 g/g meat). After 96 h storage under the aforementioned conditions, samples that were treated with the same maltodextrin/2IB3MP dosages contained 1.17 × 10^7^ CFU/g and 1.8 × 10^5^ CFU/g respectively.

### Visualization of the treatment efficiency with confocal microscopy

**Fig. 3:**
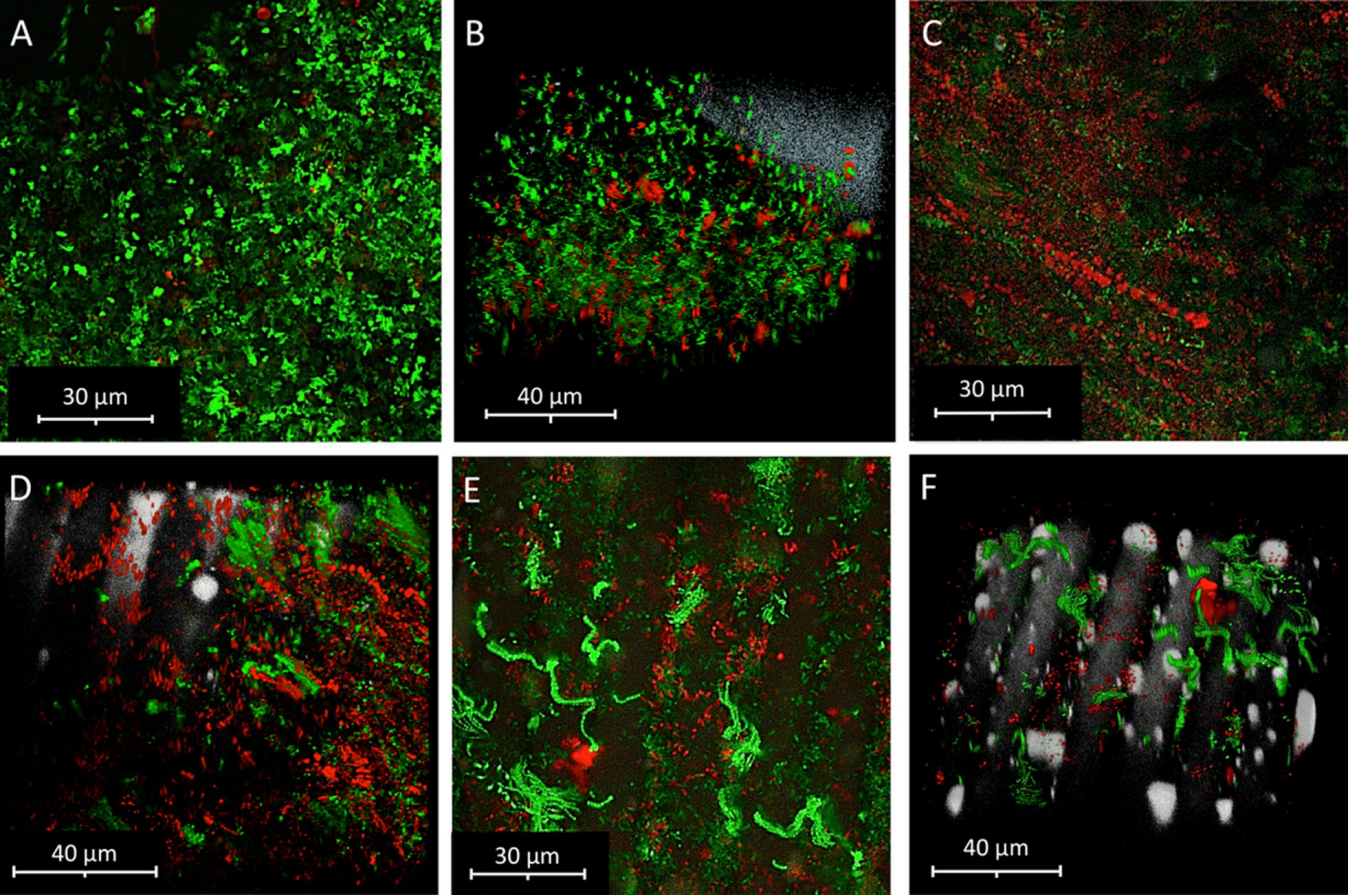
Micrographs of differential staining and confocal laser scanning microscopy. **A**, **B** Untreated controls; **C**, **D** Maltodextrin-treated samples; **E**, **F** Maltodextrin/2IB3MP-treated samples. Living bacteria are shown in green, while dead cells are depicted in red. The grey structures in the background are fibers of the textile that was used for the imprints

Contaminating bacteria that were present on the processed chicken meat were visualized with a novel textile imprint method and confocal laser scanning microscopy (CLSM). This method also includes microorganisms that were not accessible with the cultivation-dependent efficiency analyses. Micrographs with differentially stained microorganisms showed varying proportions of living and dead bacteria on the analyzed specimens (Fig. 3). Maltodextrin-treated meat strips (micrographs C and D) and maltodextrin/2IB3MP-treated chicken strips (micrographs E and F) in comparison to untreated control meat strips (micrographs A and B) showed reduced counts for living bacteria. Moreover, micrographs of the maltodextrin/2IB3MP-treated samples showed a shift in the cell morphology of the microbial population that was not observed with other samples. While the control samples primarily showed free-living cells and small aggregates, these samples harbored high proportions of chain forming microorganisms. In the samples that were only treated with maltodextrin, the total number of cells was comparable with the control. Nevertheless, the maltodextrin treatments resulted in a substantially higher proportion of dead cells.

## Discussion

### Applicability of alkylpyrazines as preservatives for processed meat products

To our knowledge, this is the first study to demonstrate preserving effects of alkylpyrazine derivatives in combination with processed meat. The utilized model substance, 2-isobutyl-3-methylpyrazine, was identified as a potential preservative, which could increase the shelf life of processed chicken meat in future applications. Efficacies of various dosages were verified in a double evaluation that combined cultivation-dependent techniques and differential staining microscopy. The overall results indicate that the combination of an antimicrobial alkyl pyrazine and maltodextrin is a promising strategy to reduce microbial contaminations on convenience products. Although no protective atmosphere was used for re-packaging of the treated meat, we observed bacterial counts that are acceptable by current regulations in the European Union (Regulation (EC) No 2073/2005). It can be expected that the efficiency of the presented method can be improved when packaging of the product is done in an industrial environment that provides further means for the avoidance of contaminations. Recent studies indicate that various natural substances, especially essential oils, can be used to increase the shelf life of chicken meat (Fratianni et al. 2010; Ntzimani et al. 2010). However, consumers’ demands are not always fulfilled by these preservation methods due to alterations in the product’s overall appearance. Here, pyrazine-rich extracts provide a promising alternative that can be applied together with maltodextrin or other suitable carriers on ready-to-eat poultry products. In addition, the application of these extracts could enhance taste properties by adding different notes of roasted flavors to the final product.

Efficiency testing in fluid pyrazine suspensions showed a high susceptibility towards the antimicrobial compound of most of the analyzed bacteria. However, isolates that were assigned to the genus *Bacillus* were not log-4 reduced by 5IB23DMP irrespective of the employed concentration. The tolerance that was observed during the efficiency testing might arise due to the presence of various pyrazines as natural metabolites in *Bacillus* spp. (Besson et al. 1997; Kosuge and Kamiya 1962). Moreover, the taxonomic group *Bacillus* is also closely related to the genus *Paenibacillus*, which includes different producers of highly effective pyrazines (Rybakova et al. 2016). Complementary imaging with confocal laser scanning microscopy (CLSM) indicated that the composition of the microbial community changes following the treatment with maltodextrin/2IB3MP. A similar phenomenon was observed when the surface of eggshells was treated with volatilized pyrazines (Kusstatscher et al. 2017). The eggshell microbiome was assessed with high-throughput sequencing of bacterial marker genes to evaluate shifts in the community structure upon decontaminations. Several pyrazine-tolerant microbes were shown to resist the treatment at lower concentrations and to increase in their relative abundance. This effect disappeared when the pyrazine concentration was increased. From the current perspective, sufficient concentrations of alkylpyrazines can substantially decrease the number of viable contaminants. For this reason, follow-up studies will focus on pyrazine-rich extracts obtained from bell peppers and other vegetables for non-conventional and natural shelf life extension.

### A new method to visualize microbial contaminants on the meat surface

To assess the treatment efficacy beyond aerobic mesophilic plate counts, a complementary approach was developed to omit cultivation-related biases. The combination of microbiological, molecular, and differential imaging experiments was highly useful in previous studies that focused on antimicrobial effects of alkylpyrazines (Cernava 2012; Kusstatscher et al. 2017). In the current study, microbial contaminations were not accessible for visualizations with CLSM imaging and differential staining due to fluorophore interferences with the meat. The developed imprint method allows to omit this unwanted interference and provides quick estimates on the preservation efficacy. Studies that focused on the applicability of live/dead staining techniques, demonstrated that the results are influenced by various physiological and chemical factors (Netuschil et al. 2014). Especially cells that are embedded in biofilms are difficult to evaluate with such approaches. Therefore, it remains to be determined how the imprint method performs when compared to in situ visualizations. Taken together, we could show with different methods that the application of alkylpyrazines substantially decreases the number of viable microorganisms on treated products. This provides the basis for future applications of these bacteria-derived metabolites that are already present in various foods that are regularly consumed by humans.

## Abbreviations

2IB3MP: 2-isobutyl-3-methylpyrazine
5IB23DMP: 5-isobutyl-2,3-dimethylpyrazine
CFU: colony forming units
CLSM: confocal laser scanning microscope
FAO: Food and Agriculture Organization of the United Nations
FEMA: Flavor and Extract Manufacturers Association
GRAS: generally recognized as safe
mVOCs: microbial volatile organic compounds
NA: nutrient agar
OECD: Organization for Economic Co-Operation and Development
